# A longitudinal study of the role of *Dichelobacter nodosus* and *Fusobacterium necrophorum* load in initiation and severity of footrot in sheep

**DOI:** 10.1016/j.prevetmed.2014.03.004

**Published:** 2014-07-01

**Authors:** Luci A. Witcomb, Laura E. Green, Jasmeet Kaler, Atiya Ul-Hassan, Leo A. Calvo-Bado, Graham F. Medley, Rose Grogono-Thomas, Elizabeth M.H. Wellington

**Affiliations:** a School of Life Sciences, University of Warwick, Gibbet Hill Campus, Coventry, UK; b School of Veterinary Sciences, University of Bristol, Langford House, Langford, UK; c UCL School of Pharmacy, University College London, 29–39 Brunswick Square, London, UK; d School of Veterinary Medicine and Science, University of Nottingham, Sutton Bonington Campus, Leicestershire, UK

**Keywords:** *Dichelobacter nodosus*, *Fusobacterium necrophorum*, Footrot, Sheep, Longitudinal study, Quantitative PCR

## Abstract

Footrot is an infectious bacterial disease of sheep that causes lameness. The causal agent is *Dichelobacter nodosus*. There is debate regarding the role of *Fusobacterium necrophorum* in disease initiation. This research used an observational longitudinal study of footrot, together with quantitative PCR (qPCR) of bacterial load of *D. nodosus* and *F. necrophorum*, to elucidate the roles of each species in the development of disease. All feet of 18 *a priori* selected sheep were monitored for five weeks assessing disease severity (healthy, interdigital dermatitis (ID) and severe footrot (SFR)) and bacterial load. A multinomial model was used to analyse these data.

Key unadjusted results were that *D. nodosus* was detected more frequently on feet with ID, whereas *F. necrophorum* was detected more frequently on feet with SFR. In the multinomial model, ID was associated with increasing log_10_ load of *D. nodosus* the week of observation (OR = 1.28 (95% CI = 1.08–1.53)) and the week prior to development of ID (OR = 1.20 (95% CI = 1.01–1.42). There was no association between log_10_ load^2^ of *F. necrophorum* and presence of ID (OR = 0.99 (95% CI = 0.96–1.02))). SFR was associated with increasing log_10_ load of *D. nodosus* the week before disease onset (OR = 1.42 (95% CI = 1.02–1.96)) but not once SFR had occurred. SFR was positively associated with log_10_ load^2^ of *F. necrophorum* once disease was present (OR = 1.06 (95% CI = 1.01–1.11)). In summary, there was an increased risk of increasing *D. nodosus* load the week prior to development of ID and SFR and during an episode of ID. In contrast, *F. necrophorum* load was not associated with ID before or during an episode, and was only associated with SFR once present. These results contribute to our understanding of the epidemiology of footrot and highlight that *D. nodosus* load plays the primary role in disease initiation and progression, with *F. necrophorum* load playing a secondary role. Further studies in more flocks and climates would be useful to confirm these findings. This study identifies that *D. nodosus* load is highest during ID. This supports previous epidemiological findings, which demonstrate that controlling ID is the most effective management strategy to prevent new cases of ID and SFR.

## 1 Introduction

Footrot is an infectious bacterial disease of sheep, which causes lameness. It is an important disease in all countries with large sheep industries. Footrot reduces sheep welfare, productivity and profitability (Egerton *et al.*, 2004; Nieuwhof and Bishop, 2005; Wassink *et al.*, 2010a). Footrot is characterised by two distinct pathological presentations: inflammation of the interdigital skin, interdigital dermatitis (ID) and separation of the hoof horn from the sensitive underlying tissue, severe footrot (SFR). Damage to the epithelium of the interdigital skin is a prerequisite for the initiation of disease (Beveridge, 1941). Spread of disease between sheep occurs when environmental conditions are conducive for indirect transmission of bacteria between sheep via pasture or pen floor (Whittington, 1995; Green and George, 2008).

ID and SFR have been treated as separate diseases in many countries in Europe, including the UK (Winter, 2008), with many veterinarians and farmers viewing ID as non-infectious and caused primarily by environmental factors, such as weather and pasture quality (Wassink *et al.*, 2005). In the UK, there is now strong evidence that that ID and SFR are two clinical presentations of the same disease (Wassink *et al.*, 2003, 2010b; Moore *et al.*, 2005). In Australia, ID and SFR have been considered one disease for many years with ID called benign footrot (scores 1–2) and SFR called virulent footrot (scores 3–4) (Egerton and Roberts, 1971; Raadsma and Dhungyel, 2013). There is some, but not complete, correlation between severity of clinical presentation of footrot and virulence traits of *D. nodosus* in Australia (Rood *et al.*, 1996; Cheetham *et al.*, 2006) and between countries (Calvo-Bado *et al.*, 2011a). However, within the UK, 300/305 isolates of *D. nodosus* from cases of ID and SFR were virulent (Moore *et al.*, 2005) indicating that virulence does not correlate with disease severity on commercial farms in the UK. Disease pathogenesis may also be affected by a range of non-bacterial factors, including host immunity and heritability of resistance traits (Escayg *et al.*, 1997) and environmental conditions, such as temperature, rainfall and pasture quality (Whittington, 1995; Wassink *et al.*, 2005).

In 1941, Beveridge produced his seminal work on footrot in which he provided evidence that *D. nodosus*, a Gram-negative anaerobe, was the primary aetiological agent of footrot rather than *Fusobacterium necrophorum*. Several decades later, it was postulated that the presence of *F. necrophorum*, a commensal of the alimentary tract of both humans and animals, was essential for development of footrot (Roberts and Egerton, 1969). Since then, Koch's molecular postulates have provided crucial evidence that the causative agent of footrot is *D. nodosus* (Kennan *et al.*, 2001, 2010). Despite these findings, *F. necrophorum* is still frequently a topic of discussion in footrot literature and is reported to be the cause or associated with both ID and/or SFR (Bennett *et al.*, 2009; Zhou *et al.*, 2009). A number of authors have investigated the presence of *D. nodosus* and *F. necrophorum* in sheep with healthy and diseased feet. *D. nodosus* is recovered more frequently from feet with ID or SFR than healthy feet (Moore *et al.*, 2005; La Fontaine *et al.*, 1993; Bennett *et al.*, 2009). Calvo-Bado *et al.* (2011b) detected *D. nodosus* on all feet of sheep using nested PCR, in a flock that had not had ID or SFR for 10 years. *F. necrophorum* was detected more frequently in feet with SFR (Bennett *et al.*, 2009). However, these studies were cross-sectional and so cause and effect could not be elucidated.

Investigation of bacterial load from uncultured material is common in ecological microbiology because culture can select for certain species of bacteria and so can introduce bias (Amann *et al.*, 1995). Such an approach is currently under-utilised in veterinary epidemiology but it can be used to improve our understanding of the process of infection and disease when culture is unreliable. This approach might inform on aetiology, pathogenesis and control of infectious diseases. *D. nodosus* is difficult to culture and PCR is more sensitive than isolation (Moore *et al.*, 2005). In addition, given that Calvo-Bado *et al.* (2011b) reported that *D. nodosus* was detectable in all feet using nested PCR, irrespective of disease state, load of *D. nodosus* might be a more useful tool to investigate the role of *D. nodosus* and *F. necrophorum* in the pathogenesis of footrot. Quantitative PCR (qPCR) is used to determine bacterial load. Key features that are required for accurate qPCR analyses include a specific sequence (amplicon) present in all strains of only the target bacterial species, a low limit of detection (analytical sensitivity) and no cross reactivity with other non-target microorganisms (analytical specificity).

The aim of this paper was to use sensitive and specific qPCR assays to investigate the change in load of *D. nodosus* and *F. necrophorum* in feet and sheep developing ID and SFR and to elucidate the temporal patterns between bacterial load and disease and so identify the roles of *D. nodosus* and *F. necrophorum* in disease initiation and progression.

## 2 Materials and methods

### 2.1 Study population

The study flock comprised of 570 Mule, Suffolk and Roussin ewes. The flock was located on a lowland farm in Oxfordshire, England with a mean rainfall of 10–20 mm per month and a mean daily temperature of 11 °C. The study was done in September/October 2006 when environmental conditions (rainfall and temperature) were conducive for transmission of disease. The flock had had lame sheep with SFR for >20 years, with a prevalence of 6–8% lameness at any one time (Wassink *et al.*, 2010a). During the current study 30.5% of sheep in the flock had ID and 4.7% of sheep had SFR.

### 2.2 Sample collection and disease severity scoring

From this flock a subset of 60 sheep were selected (Kaler *et al.*, 2011). All 4 feet of all 60 ewes were examined each week for 5 weeks. Each foot was recorded as clinically healthy, having ID or having SFR using a defined system (Foddai *et al.*, 2012) and then the interdigital skin was swabbed by a single trained researcher (JK), in order to standardise the sampling method and to avoid between observer variation. All swabs were collected and stored in transport buffer at −80 °C until required (Moore *et al.*, 2005). The study was approved by the University's local ethical committee. From these 60 sheep, 12 sheep were purposively selected for the current study, 3 ewes with no signs of disease during the 5 week study, 5 ewes with ID but no SFR and 4 ewes with SFR (with or without ID). An additional 6 ewes (2 with ID but no SFR and 4 with SFR) were then selected and their samples analysed to test the consistency of the initial results (Table 1) giving 360 observations of 72 feet from 18 ewes. A total of 100 feet had ID and 24 feet had SFR.

### 2.3 Sample processing

Swab samples were thawed, vortexed briefly to suspend swabbed material and the swab then removed from the liquid sample. Chromosomal DNA was extracted from a 200 μl liquid aliquot using the NucleoSpin Blood kit (Macherey-Nagel, ABgene, Epsom, UK) and stored at −20 °C, as done previously (Moore *et al.*, 2005).

### 2.4 Primer and probe design

Two TaqMan^®^ qPCR assays were designed using the Primer Express^®^ software (v.3.0) (Applied Biosystems, Foster City, California, USA), the first targeted a 61 bp sequence within the *rpoD* gene (RNA polymerase sigma subunit) of *D. nodosus* (Calvo-Bado *et al.*, 2011b). The second novel assay targeted an 86 bp sequence within the *rpoB* gene (RNA polymerase beta subunit) of *F. necrophorum* subsp. *necrophorum* (accession no. AF527637.1); forward primer 5′-AAC CTC CGG CAG AAG AAA AAT T-3′, reverse primer 5′-CGT GAG GCA TAC GTA GAG AAC TGT-3′ and TaqMan^®^ probe 5′-6FAM-TCG AAC ATC TCT CGC TTT TTC CCCGA-BBQ-3′. The *F. necrophorum* genome has not yet been sequenced and the calculation for the standards for the *rpoB* assay relies on the assumption that it is of a similar molecular size to the sequenced *F. nucleatum* subsp. *nucleatum* genome (accession no. NC_003454) and that *F. necrophorum* also contains a single copy of the *rpoB* gene per cell (Aliyu *et al.*, 2004). Primer and probe sets were selected based on the low penalty score and low amplicon size allocated by the software. BLAST analysis was performed for the *rpoB* target sequence. The *F. necrophorum* (*rpoB*) primer and probe set were designed to target both subspecies; *F. necrophorum* subsp. *necrophorum* and subsp. *funduliforme*. Primer and probe sets were synthesised and purified commercially (TIB MOLBIOL, GmbH, Berlin, Germany). The probes were labelled at the 5′-end with the fluorescent dye FAM (6-carboxyl-fluorescein) and at the 3′-end with the non-fluorescent quencher BBQ (Black Berry Quencher).

### 2.5 qPCR standard curve preparation

*D. nodosus* (VCS1703A) was cultured as described previously (Calvo-Bado *et al.*, 2011b) and *F. necrophorum* (clinical isolate BS-1) was grown on Wilkins-Chalgren anaerobe agar (Oxoid, Basingstoke, UK) at 37 °C for 48 h. Chromosomal DNA was isolated from cells using the NucleoSpin Blood kit (Macherey-Nagel) according to the manufacturer's instructions and then quantified using the NanoDrop^®^ (ND-1000) spectrophotometer (Labtech International Ltd., Luton, UK). Serial dilutions of *D. nodosus* and *F. necrophorum* DNA were then prepared to provide an estimated 2.04 × 10^6^ to 2.04 genome copies μl^−1^ and 2.47 × 10^7^ to 2.47 genome copies μl^−1^, respectively.

### 2.6 qPCR cycling conditions

The qPCR assays were performed as previously described (Calvo-Bado *et al.*, 2011b), except that the annealing temperature for the *F. necrophorum* (*rpoB*) assay was increased to 61 °C, in order to eliminate non-target detection (data not shown). If no increase in the fluorescence signal was observed after 40 cycles, the sample was defined as ‘undetectable’ (below the limit of detection (LOD)).

### 2.7 qPCR analytical specificity and sensitivity

The analytical specificity of the *D. nodosus* (*rpoD*) qPCR assay has previously been described (Calvo-Bado *et al.*, 2011b). For the *F. necrophorum* (*rpoB*) qPCR assay a total of 14 *F. necrophorum* (consisting of both subspecies) strains and 15 negative controls were screened (see supplementary file). PCR amplicon size and specificity (production of a single band) was determined using gel electrophoresis (3% (w/v) agarose). PCR products from both assays were cloned into the TOPO 2.1 vector system (Invitrogen, Ltd., Paisley, UK) and the inserts were sequenced and aligned with sequences available in GenBank.

The theoretical detection limit (TDL) for both assays was determined by setting up a series of spiking experiments. Sterile cotton swabs were submerged into 0.5 ml phosphate buffered saline (PBS) containing 20 mM Na_2_EDTA (pH 8.0) (Moore *et al.*, 2005). Swabs were spiked with 0.5 ml undiluted and serially diluted *D. nodosus* (VCS1703A) culture and 0.5 ml of each of the seven 10-fold serial dilutions (10^−1^–10^−7^), resulting in approximately 9.20 × 10^8^ to 9.20 × 10^1^ *rpoD* copies swab^−1^. Additional swabs were then spiked with 0.5 ml undiluted *F. necrophorum* (clinical isolate BS-1) culture and 0.5 ml of each of the seven 10-fold serial dilutions (10^−1^ - 10^−7^), resulting in approximately 3.75 × 10^7^ to 3.75 *rpoB* copies swab^−1^. The TDL was then estimated using the calculation stated by Pontiroli *et al.* (2011). Finally, swabs that were negative for both *D. nodosus* and *F. necrophorum* after sampling the interdigital skin were also spiked with 10^−1^ and 10^−2^ dilutions of *D. nodosus*and *F. necrophorum* culture to determine whether skin exudate/contaminating material (e.g. soil/faecal matter) present on the swabs interfered (inhibited) with the PCR reaction (see supplementary file).

### 2.8 Statistical analysis

Data were summarised by presence/absence and mean log_10_ *D. nodosus* (*rpoD*) and *F. necrophorum* (*rpoB*) load (+1) by disease state (healthy, ID, SFR) of feet (Table 2).

An unordered multinomial mixed effects model accounting for repeated observations and samples of over time clustered within ewes was used to examine the associations between *D. nodosus* and *F. necrophorum* presence and load by disease status over time in MLwiN 2.21 (Rasbash *et al.*, 2005). The outcome variable had three categories; healthy, ID and SFR affected feet. The explanatory variables were week of study (categorical), foot (categorical), *D. nodosus* and *F. necrophorum* log_10_ (load +1) (+1 resulted in undetectable load coded as 0 on a log scale) and polynomials of load at an observation and lagged (week prior to the observation) (presented as lag log_10_ load). The model was built using a stepwise (both forward stepwise and backward elimination) approach until no variables added increased the model fit and no variables removed reduced model fit. The equation took the form:

$$\text{Log}(\pi_{1\text{jk}/\text{pi}0\text{jk}})=\beta_{0\text{k}}+\sum \beta_{0}x_{\text{jk}}++\sum \beta_{0}x_{\text{j}}+v_{0\text{k}}$$

$$\text{Log}(\pi_{2\text{jk}/\text{pi}0\text{jk}})=\beta_{1\text{k}}+\sum \beta_{1}x_{\text{jk}}++\sum \beta_{1}x_{\text{j}}+v_{1\text{k}}$$

where log(*π*_1jk/pi0jk_) = the probability of ID versus healthy and log(*π*_2jk/pi0jk_) = the probability of SFR versus healthy, *β*_0k_ and *β*_1k_ are constants for ID and SFR, *β*_0_*x* and *β*_1_*x* are vectors of fixed effects for ID and SFR varying at level 1 and 2, where level 1(j) = week and level 2(k) = sheep, where *v*_0k_ and *v*_1k_ are level 2 residual variances and level 1 is assumed to take a binomial error distribution. The model was developed using RIGLS (restricted unbiased iterative generalised least squares) and then MCMC was used to adjust for the possibility of overinflated standard errors. A burn-in of 5000 followed by 50,000 iterations was done. Significance was determined using the Wald's statistic, where 95% CI did not include unity. The model fit was tested by outputting the predictions from the model and comparing sum ranked fitted quintile estimates against the summed observations for the number of cases of ID and SFR combined each week using the Hosmer Lemeshow test (Dohoo *et al.*, 2003). The model was rerun with all undetectable loads omitted.

## 3 Results

### 3.1 qPCR assay performance

The TDL for both qPCR assays was approximately 10^3^ *rpoD* and *rpoB* copies swab^−1^. There was no significant difference in TDL in swabs with and without lesion exudate (see supplementary file). The calibration standards for both the *D. nodosus* and *F. necrophorum* assays generated *R*^2^ values of ≥0.995 (Pearson's coefficient for determination) and mean slope values of −3.6 (SEM ± 0.04) and −3.7 (SEM ± 0.04) (PCR efficiency), respectively, indicating high amplification efficiencies.

The specificity of the *D. nodosus* qPCR assay is published elsewhere (Calvo-Bado *et al.*, 2011b). The novel *F. necrophorum* qPCR assay amplified all *F. necrophorum* isolates screened and did not cross-react with non-target microorganisms (see supplementary file). The *rpoB* amplicons produced a single discrete band of the expected size (86 bp) and cloned sequences matched the GenBank sequences (Witcomb, 2012), indicating that the assay was specific to *F. necrophorum*.

### 3.2 Detection of D. nodosus and F. necrophorum from ovine foot swabs by qPCR

There were 349 swabs where DNA was extracted; 225 swabs from healthy feet, 100 swabs from feet with ID and 24 swabs from feet with SFR (Table 1). *D. nodosus* was detected on 68.4% of healthy feet, 86.0% of feet with ID and 70.8% of feet with SFR. *F. necrophorum* was detected on 62.2% of healthy feet, 64.0% of feet with ID and 75.0% of feet with SFR.

There were 3 sheep (54 foot swabs) that were healthy throughout the 5-week study, 7 sheep (138 foot swabs) where at least one foot per sheep had ID but no SFR and 8 sheep (157 swabs) where at least one foot per sheep had SFR. *D. nodosus* was detected on all sheep at all time points with the exception of one (healthy) sheep where *D. nodosus* was not detected on any feet in weeks 2–5. *F. necrophorum* was detected on all sheep at all time points with the exception of three different sheep, each with one time point when *F. necrophorum* was not detected. *D. nodosus*was detected on 42.6% (23/54) swabs from healthy sheep, from 84.8% (117/138) swabs from sheep with only ID and from 75.2% (118/157) swabs from sheep with SFR. *F. necrophorum* was detected on 58.9% (28/54) swabs from healthy sheep, 64.9% (89/138) swabs from sheep with ID only and 68.2% (107/157) swabs from sheep with SFR.

### 3.3 Multinomial mixed regression model

The log_10_ mean load of *D. nodosus* and *F. necrophorum* by week and disease status is presented in Table 2. Data were then analysed using an unordered multinomial mixed regression model (Table 3). Compared with a baseline of healthy feet, there was a significant increased risk (OR = 1.28 (95% CI = 1.08–1.53)) of ID with increasing *D. nodosus* load the week ID was present, indicating that load increased during an episode of ID. In addition, there was a significant association between increased *D. nodosus* load the week before ID was present (OR = 1.20 (95% CI = 1.01–1.42)). There was a significant association between load of *D. nodosus* and SFR, the week before SFR was present (OR = 1.42 (95% CI = 1.02–1.96)). SFR was positively associated with the quadratic of load of *F. necrophorum* the week of disease (OR = 1.06 (95% CI = 1.01–1.11)), however there was no association between load of *F. necrophorum* and ID. There was an increased risk of ID and SFR in weeks 4 and 5 of the study. From the random terms, there was variation and covariation between healthy, ID and SFR feet at the level of sheep (Table 3). The model fit was good with predicted values non-significantly different from observed values by week of observation. Data from 12 sheep only gave similar results. The data from all 18 sheep were rerun with all undetectable estimates for *D. nodosus* and *F. necrophorum* omitted and gave similar results with coefficients slightly different, but with the same significance.

## 4 Discussion

In this study we have elucidated the temporal patterns of presence and load of*D. nodosus* (*rpoD*) and *F. necrophorum* (*rpoB*) in relation to the presentation and development of ID and SFR in 18 sheep. The key findings are that *D. nodosus* load increases significantly before and during an episode of ID and prior to the occurrence of SFR, whilst *F. necrophorum* load is only higher in feet once SFR had occurred. These results indicate that an increase in *D. nodosus* load drives the pathogenesis of footrot whilst *F. necrophorum* is a secondary invader.

The mean log_10_ *D. nodosus* load was highest in feet with ID compared with feet with SFR and feet that were healthy (Table 2). In addition, increased load of *D. nodosus* was present before and during an episode of ID (Table 3), indicating that *D. nodosus* was driving the early stages of footrot. We therefore hypothesise that if load is indicative of infectiveness, sheep with ID are likely highly infective and are also likely to be more infective than sheep with SFR. This is consistent with the finding that a high prevalence of ID is associated with an increased risk of development of more cases of ID and SFR in subsequent weeks (Green *et al.*, 2007) and supports empirical evidence that rapid treatment of both sheep with ID and SFR (Wassink *et al.*, 2010a,b) and separation of sheep with ID from the main flock (Wassink *et al.*, 2003) reduces the incidence of ID and SFR. These results highlight a need to change current perception of ID (footrot scores 1–2) among researchers, veterinarians and farmers in many countries where footrot is endemic, to target disease control at this early stage of the disease process.

Whilst there was an association between SFR and increasing *D. nodosus* load the week before disease onset, there was no significant association between SFR and *D. nodosus* load once SFR was present. This result is consistent with previous studies where only detection was studied (Moore *et al.*, 2005; Calvo-Bado *et al.*, 2011b) and a study of *D. nodosus* in foot biopsies using fluorescence *in situ* hybridisation (FISH) (Witcomb, 2012). The reason for this reduction in load is not known, but we postulate that (i) the majority of microorganisms may be removed by the sloughing of necrotic epithelial tissue (Beveridge, 1941; Roberts and Egerton, 1969; Witcomb, 2012), (ii) that secondary invaders outcompete *D. nodosus* by this stage or that (iii) separation of the hoof exposes the deeper layers of the foot to increased levels of oxygen, resulting in the decline of this anaerobic species.

An increase in *F. necrophorum* load^2^ was only observed once SFR had developed. It is possible that *F. necrophorum* load increased before SFR occurred but after the previous weekly swab sample was collected. The development from ID to SFR occurred in the 1–6 days after the previous sampling and so the rate of change in load would have to have been very rapid and faster than that for *D. nodosus.* In addition, the experimental evidence that *D. nodosus* causes SFR is compelling from Beveridge (1941) to Kennan *et al*., (2001, 2010).

Our results suggest that *F. necrophorum* plays an opportunistic, or secondary role, once footrot has developed rather than a causal role in disease initiation. This is consistent with understanding of the role of *Fusobacterium* spp. in other diseases. *F. necrophorum* and other *Fusobacteria* are present in lesions and abscesses in many polymicrobial infections (Brook and Frazier, 1997; Brook, 2002; Hofstad, 2006), where they are considered to enhance disease severity through synergistic relationships with other pathogens (Brook and Walker, 1986; Tan *et al.*, 1996). Whether or not *F. necrophorum* enhances footrot severity has yet to be elucidated. *F. necrophorum* is a commensal in the alimentary tract and shed, at least on occasion, in faeces (Roberts and Egerton, 1969; Smith and Thornton, 1997), consequently *F. necrophorum* is present in lesions of sheep feet, whatever the causal pathogen.

This was a small prospective study of a UK flock with footrot endemic in the flock for many years. The epidemiological data were collected by one experienced observer (JK) using a validated scoring system (Foddai *et al.*, 2012) to avoid between observer bias. Swabs were taken using standard procedures and one trained laboratory researcher (LAW) processed and analysed all the samples. Swabs are frequently used to obtain DNA for qPCR analysis in clinical research (Fredricks *et al.*, 2009; Srinivasan *et al.*, 2010; Koren *et al.*, 2011) and they are typically used to sample ovine interdigital skin (Moore *et al.*, 2005; Bennett *et al.*, 2009; Hill *et al.*, 2010). Swabs are non-invasive, which is essential for longitudinal sampling where punch biopsies would cause damage and, as well as ethical issues, would change the natural course of disease. The recovery of material from swabs could have been variable in the current study, however, sampling precision was increased by having a single trained operator collecting the swabs and a second analysing the swabs. Unfortunately within foot reliability cannot be tested because replicate swab samples will have a reduced load (Chamberlain *et al.*, 1997). qPCR may overestimate bacterial load due to the detection of DNA from lysed cells, however, similar changes in both the *D. nodosus* and *F. necrophorum* populations by disease status were also observed *in situ* using FISH (Witcomb, 2012), providing further evidence for the relative abundance of these two species and their associations with ID and SFR.

The statistical associations between disease status and bacterial load also indicate that the swabbing method was robust. Had random numbers of bacteria been detected per swab there would have been no statistical association between load and disease because of misclassification caused by random error. Re-analysing the data with undetectable loads missing gave similar results, indicating that undetectable loads were not random but were lower loads of bacteria. An unordered multinomial model was used because previous work suggested that load does not increase with disease development from ID to SFR (Moore *et al.*, 2005) and so no order in relationship between disease severity and load was assumed. Ideally an *a priori* sample size calculation would have been done, however, there were no studies on the likely load or variation in load to estimate a sample size. Consequently 12 sheep with a range of clinical presentations were analysed initially and then, to add robustness a further 6 selected sheep were analysed and the statistics repeated. Results were similar for both 12 and 18 sheep. Whilst this was a small number of sheep to study, the results were statistically significant and consequently sufficient for our conclusions and provides a baseline for future studies. Given the consistency of our results with previous literature, we consider that the results and inferences are likely to be generalisable to other flocks with endemic footrot, particularly those areas with climates similar to the UK. However, we have yet to determine whether the results are consistent globally and further work on several flocks in different climates and with different virulence patterns of *D. nodosus* strains would be highly informative.

## 5 Conclusions

This is the first study to examine *D. nodosus* and *F. necrophorum* load over time and we have demonstrated the importance of *D. nodosus* in the development and presence of ID and progression to SFR, whilst highlighting the opportunistic nature of *F. necrophorum*. This information provides an improved understanding of the population dynamics associated with pathogenesis of footrot and reinforces empirical studies that highlight that optimal control strategies for footrot include targeting sheep with ID, as well as those with SFR.

## Funding

L.A. Witcomb was a NERC CASE studentship with Pfizer Animal Health as the industrial partner. All other authors were supported by Combating Endemic Diseases of Farmed Animals for Sustainability (CEDFAS) initiative, Grant No. BBE01870X1 from the Biotechnology and Biological Sciences Research Council(BBSRC). Original data were collected by JK during an MLC funded studentship.

## Conflict of interest statement

The authors declare there is no conflict of interest.

## Figures and Tables

**Table 1:** Number and percent of feet that were healthy, had interdigital dermatitis (ID) or had severe footrot (SFR) among 18 sheep that remained healthy or developed ID or SFR during the study.

Sheep code	Disease status of sheep	Percent (number/20) healthy feet	Percent (number/20) feet with ID	Percent (number/20) feet with SFR (± ID)
2685^a^	Healthy	100 (20)	0 (0)	0 (0)
2720	Healthy	100 (20)	0 (0)	0 (0)
2229	Healthy	100 (20)	0 (0)	0 (0)
2705^a^	Interdigital dermatitis	85 (17)	15 (3)	0 (0)
2223	Interdigital dermatitis	80 (16)	20 (4)	0 (0)
2620	Interdigital dermatitis	75 (15)	25 (5)	0 (0)
2274	Interdigital dermatitis	65 (13)	35 (7)	0 (0)
2314^a^	Interdigital dermatitis	60 (12)	40 (8)	0 (0)
2301	Interdigital dermatitis	60 (12)	40 (8)	0 (0)
2208	Interdigital dermatitis	40 (8)	60 (12)	0 (0)
2234	Severe footrot	40 (8)	55 (11)	5 (1)
2211	Severe footrot	50 (10)	45 (9)	5 (1)
2610^a^	Severe footrot	70 (14)	15 (3)	15 (3)
2225	Severe footrot	30 (6)	55 (11)	15 (3)
2714^a^	Severe footrot	25 (5)	60 (12)	15 (3)
2290	Severe footrot	70 (14)	10 (2)	20 (4)
2613	Severe footrot	65 (13)	15 (3)	20 (4)
2650^a^	Severe footrot	55 (11)	20 (4)	25 (5)

a 11/360 swab samples missing.

**Table 2:** Mean log_10_ (*D. nodosus* (Dn) load + 1) and mean log_10_ (*F. necrophorum* load (Fn) + 1) (*rpoD*/*rpoB* copies swab^−1^) by weeks 1–5 and by disease status of feet (healthy, interdigital dermatitis (ID) and severe footrot (SFR)), 349 observations of 18 sheep. For all feet^a^ and for feet with detectable load only.^b^

	Status of foot	Number of feet	Log_10_ Dn/Fn	Number of feet with detectable Dn/Fn	Log_10_ Dn/Fn positive feet	Log_10_ Dn/Fn by week				
						Week 1	Week 2	Week 3	Week 4	Week 5
Dn	Healthy	225	3.14	154	4.56	3.67	3.20	3.28	2.14	2.97
	ID	100	4.42	84	5.14	4.57	3.81	3.69	4.25	5.08
	SFR	24	3.55	17	5.02	4.05	5.09	3.11	3.25	2.80

Fn	Healthy	225	2.82	141	4.48	4.06	2.89	2.29	2.63	1.97
	ID	100	2.79	64	4.36	3.28	3.28	1.70	2.78	2.57
	SFR	24	3.74	18	4.98	5.60	4.12	2.18	4.01	3.01

a log_10_ (load +1).

b log_10_ (load) – feet with below limit of detection (LOD) coded zero on log_10_ scale.

**Table 3:** Multinomial mixed effect regression model of (log_10_ +1) *D. nodosus* and (log_10_ +1) *F. necrophorum* load in 18 sheep from one farm over weeks 5 weeks (274 observations).

Response	Interdigital dermatitis						Severe footrot					
	Univariable			Multivariable			Univariable			Multivariable		
Fixed part	OR	Lower 95% CI	Upper 95% CI	OR	Lower 95% CI	Upper 95% CI	OR	Lower 95% CI	Upper 95% CI	OR	Lower 95% CI	Upper 95% CI
Intercept												
Week_2	Baseline											
Week_3	0.48	0.16	1.44	0.44	0.13	1.43	0.76	0.16	3.61	1.75	0.31	10.05
Week_4	8.44	3.39	21.00	9.81	3.67	26.23	3.42	0.86	13.60	7.68	1.37	42.94
Week_5	6.63	2.66	16.48	6.15	2.25	16.85	2.99	0.73	12.17	6.73	1.26	36.01
Foot – left fore	Baseline											
Foot – right fore	2.33	1.06	5.15	1.15	0.36	3.63	1.06	0.31	3.68	0.22	0.03	1.45
Foot – left hind	3.75	1.73	8.15	2.59	0.83	8.07	1.71	0.54	5.49	0.39	0.07	2.37
Foot – right hind	2.82	1.29	6.20	1.92	0.65	5.67	0.54	0.12	2.47	0.14	0.02	1.13
Log_10_ Dn load +1	1.20	1.05	1.37	1.28	1.08	1.53	1.04	0.85	1.29	0.97	0.75	1.26
Lag log_10_ Dn load +1	1.23	1.11	1.37	1.20	1.01	1.42	1.13	0.94	1.36	1.42	1.02	1.96
Log_10_ Fn load +1	0.95	0.84	1.07				1.18	0.92	1.51			
(Log Fn load +1)^2^	0.99	0.97	1.01	0.99	0.96	1.02	1.04	1.00	1.08	1.06	1.01	1.11
Random part												
Variance interdigital dermatitis (ID)	2.35	0.96	2.26	1.28								
Co-variance ID/SFR	1.26	0.82	2.24	1.70								
Variance severe footrot (SFR)	2.10	1.17	5.94	5.36								

Dn = *Dichelobacter nodosus*, Fn = *Fusobacterium necrophorum*, Lag = week prior to disease onset, OR = odds ratio, CI = confidence interval, variables significant at 0.05 when CI do not include unity (Wald's test).
