# Supplementary material for: A longitudinal study of the role of Dichelobacter nodosus and Fusobacterium necrophorum load in initiation and severity of footrot in sheep
Source: Prev Vet Med. 2014 Jul 1;115(1-2):48–55. doi: 10.1016/j.prevetmed.2014.03.004 (PMC4029074; doi:10.1016/j.prevetmed.2014.03.004)
Supplement: Supplementary file 1 [file mmc1.pdf]

Supplementary Information

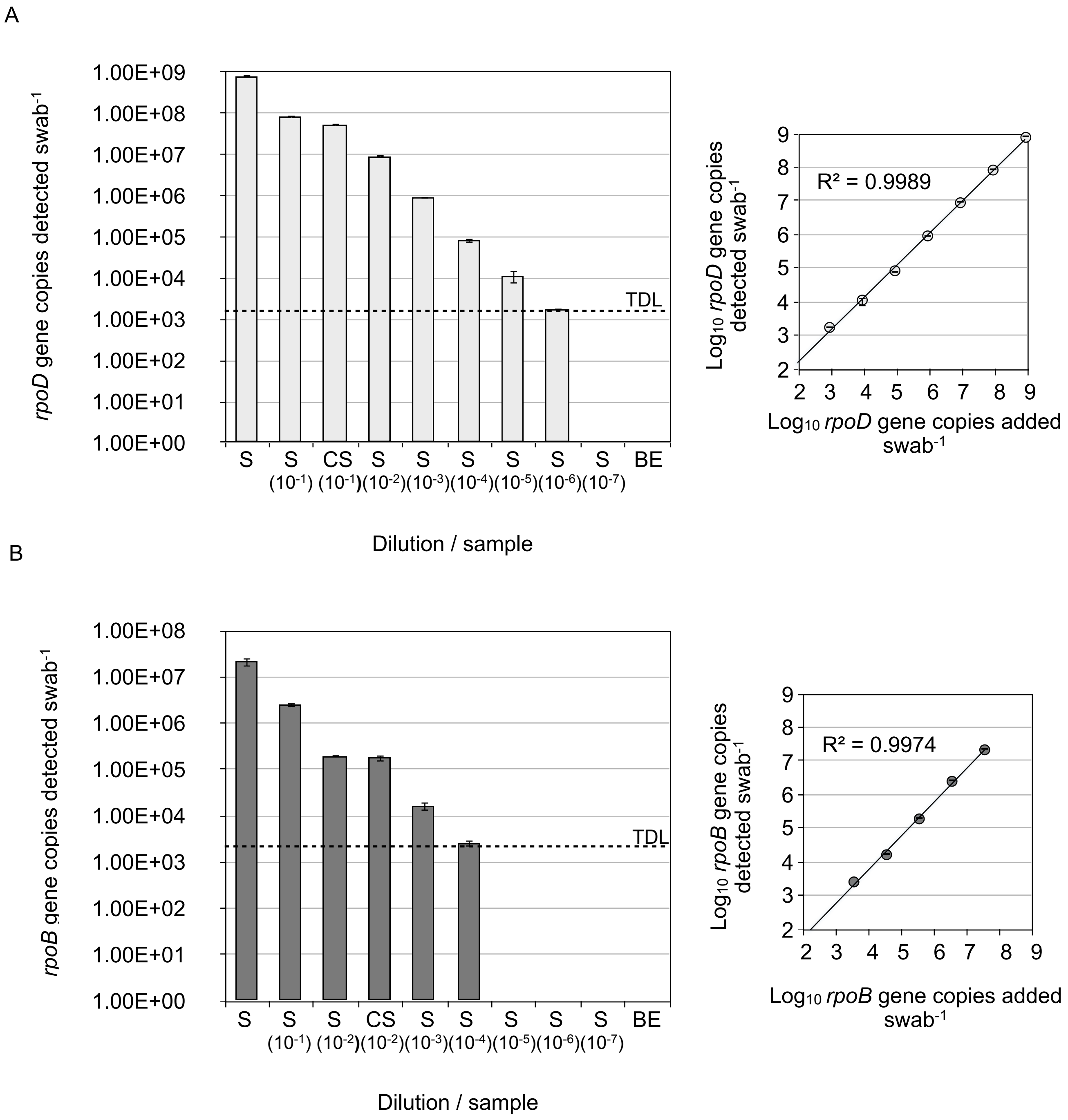

Analytical sensitivity of (A) *D. nodosus* (*rpoD*) and (B) *F. necrophorum* (*rpoB*) qPCR assays for swabs determined by spiking experiments. Data presented as last detectable dilution (S) with theoretical detection limit (TDL) indicated and $\log_{10}$ *rpoD*/*rpoB* copies detected swab^{-1} against $\log_{10}$ *rpoD*/*rpoB* copies added swab^{-1}.
